# Mutant Pattern of p53 as a Feasible Predictor of Distant Metastasis Following Curative Gastrectomy for Advanced-stage Gastric Cancer

**DOI:** 10.7150/jca.98563

**Published:** 2025-01-01

**Authors:** Quanming An, Lili Miao, Jia Wu, Junwen Ma

**Affiliations:** 1 Department of Gastrointestinal Surgery, The General Hospital of Ningxia Medical University, Yinchuan, Ningxia 750004, China.; 2 Department of Respiratory and Critical Care Medicine,The General Hospital of Ningxia Medical University, Yinchuan, Ningxia 750004, China.; 3 Department of Gastroenterology,The General Hospital of Ningxia Medical University, Yinchuan, Ningxia 750004, China.

**Keywords:** mutant pattern, p53, distant metastasis, gastrectomy, gastric cancer

## Abstract

**Objective:** The TP53 mutation is a poor prognostic factor for malignant tumors in a number of organs. The present study primarily aimed to clarify the impact of the mutant pattern of p53 on the prognosis and recurrence of gastric cancer.

**Methods**: For this purpose, 519 patients who underwent radical gastrectomy for cancer were enrolled in the present study. Immunohistochemistry (IHC) was used to examine p53 expression in tissues and a three-stage classification system was used to divide the patient tissues into three groups according to the expression of p53: Heterogeneous (wild-type), absent and overexpression (mutant).

**Results:** After 5 years of follow-up, recurrence and metastasis occurred in 38.7% of patients with stomach cancer, with a p53 mutant pattern in 48.4% of these patients. Patients with a p53 mutant pattern had lower recurrence-free and overall survival rates at 5 years compared with those who were p53 wild-type (P<0.001). It was found that the p53 pattern differed significantly (P<0.001) between the wild-type and mutant patterns, including the pN0 and pN+ gastric cancer subgroups (P<0.001 and P=0.014, respectively). The p53 mutant pattern was also significant in the determination of the recurrence-free survival of patients with progressive stomach cancer (P<0.0001). The 5-year overall survival rates were 71.7 and 36.2%, and the recurrence-free survival rates were 71.2 and 35.2% in the pN0 and pN+ groups, respectively (P<0.001). The mutant pattern of p53 was a significant prognostic factor for both distant metastasis [relative risk (RR)=2.881, P<0.001] and overall survival (RR=2.809, P<0.001) in the univariate Cox regression analysis. In the multivariate analysis, distant metastasis (RR=2.767, P<0.001) remained significant in the mutant pattern of p53 staining. After propensity score matching, 189 patients with a p53 wild-type and 189 patients with a p53 mutant pattern were extracted for analysis. The 5-year overall survival rate in patients with the p53 mutant pattern (n = 189) was worse than that in the patients with p53 wild-type (n = 189) and with significant differences (log-rank P<0.01). The study was statistically significant after Cox univariate and multivariate regression analysis, which revealed that the mutant pattern of p53 is an independent prognostic factor impacting distant metastases following curative gastrectomy for advanced-stage gastric cancer (p = 0.48).

## Introduction

Currently, stomach cancer remains a serious global health issue, particularly in East Asian nations. Gastric cancer ranked third globally in terms of cancer-related mortality in 2020, with >1 million cases worldwide, leading to > 768,000 related deaths. This renders gastric cancer the fifth most commonly diagnosed type of cancer worldwide 1. At present, the only treatment option considered to be effective for stomach cancer is radical surgery; nevertheless, the prognosis of patients following this procedure is markedly impacted by the high rates of metastasis and recurrence 2,3. Although the 5-year survival rate of early stomach cancer can reach >90%, most patients present with advanced-stage gastric cancer due to the low early diagnosis rate 4. Although improved surgical procedures and perioperative care have reduced operative mortality and morbidity, the long-term prognosis for gastric cancer is still poor. Adjuvant chemotherapy or radiochemotherapy is also less effective against than other solid tumors due to tumor biology heterogeneity. Recurrence following curative gastrectomy is the leading cause of cancer-related death, affecting 20% to 50% of patients 5. Recurrence is classified into three categories as follows: Local recurrence, peritoneal metastasis or distant metastasis. Local recurrence includes the reappearance of cancer at either the anastomotic site or the gastric stump, as well as the spread of cancer to nearby lymph nodes. The presence of peritoneal metastases has been verified through the radiology reports of patients, which indicates the presence of peritoneal nodules, or via the positive cytological analysis of ascites in their cross-sectional images. Metastatic ovarian cancer has been classified as peritoneal metastases. The categorization of distant metastases has been expanded based on the specific organ affected. Abdominal lymph nodes, excluding cervical lymph nodes and the upper retroperitoneum, have been classified as distant metastases. Multiple recurrences are defined as the occurrence of a disease in two or more sites 6,7.

For individuals with stomach cancer, tumor grading and lymph node metastases are independent predictors of prognosis 8,9. However, the clinicopathological staging of gastric cancer is not completely consistent with the biological characteristics of the tumor. Pathological staging does not fully reflect the complete prognosis of the tumor and the risk of recurrence and mortality, particularly in progressive gastric cancer. The emergence of molecular subtyping is becoming a key method for the diagnosis and treatment of stomach cancer. According to The Cancer Genome Atlas (TCGA) project 10, the clinicopathological classification of gastric cancer does not align with tumor biology. Pathological classification does not fully reflect the prognosis of the tumor or the risk of recurrence and mortality, particularly in advanced-stage gastric cancer. The development of molecular subtypes has emerged as a crucial strategy for the diagnosis and treatment of gastric cancer. Molecular markers are a critical prognostic factor for tumors. The Epstein-Barr virus (EBV)-positive, microsatellite instability (MSI), genomic stability (GS) and chromosomal instability (CIN) subtypes are the four molecular subtypes of gastric cancer. The best prognosis is linked to the EBV subtype, while the worst prognosis is linked to the GS subtype. The CIN subtype has the greatest clinical significance, as adjuvant chemotherapy has the greatest benefit for this subtype 11. In gastric cancer, TP53 is included in the CIN subtype and is one of the most crucial factors in the molecular subtypes. Tumors with a high copy number exhibit recurrent mutations affecting the TP53 gene. The TP53 controls senescence, apoptosis, DNA repair and cell cycle checkpoints, all of which are crucial for maintaining GS. Mutant TP53 loses its antitumor transcriptional activity and often acquires oncogenic functions that promote tumor cell proliferation, invasion and drug resistance 12-15.

Approximately half of all human malignancies have p53 gene mutations. p53 staining is commonly reported as either positive or negative; however, Köbel believes this is confusing terminology; patterns of staining, which should be reported as wild-type or aberrant/abnormal/mutant, etc., are described. Researchers recently 16,17 used a three-tiered system of immunohistochemistry (IHC) to study p53. Overexpression and complete absence were observed as mutant types, while the level of p53 expression between these extremes was considered to be wild-type. The three observed patterns were the following: i) Heterogeneity (wild-type), with varying intensity and percentage of nuclear staining; ii) overexpression, with diffuse and strong staining in the majority of tumor cells; and iii) absence of staining, with the majority of tumor cells being unstained, with wild-type staining exhibiting a mixture of negative, weak-positive and strong-positive cells. Overexpression and complete absence are characterized by diffuse or minimal nuclear staining, as opposed to normal/wild type. There have been studies on ovarian cancer that have demonstrated that optimized IHC agrees very well with this (up to 100% specificity) when the mutant p53 status is present 10-12. In other words, in the case that the p53 staining pattern is abnormal (aberrant/mutant phenotype), an underlying mutant p53 is almost certainly present.

IHC of the p53 protein can be performed in the majority of pathology laboratories. It is easily performed on gastric cancer biopsy samples. It can also help to determine adjuvant chemotherapy, and the timing of recurrence and survival. In this context, we aimed to identify the clinicopathologic significance of the p53 expression pattern on IHC using a three-tiered system in patients undergoing radical surgery for stomach cancer. It is of critical importance clinically to determine methods through which to examine and understand key prognostic factors, and to develop strategies with which to combat recurrence and metastasis in order to improve the prognosis of patients. This can only be achieved by examining the clinicopathological data of patients with gastric cancer in extensive detail.

## Patients and Methods

### Patients

The present study was authorized by the General Hospital of Ningxia Medical University's Ethics Committee (approval number: KYLL-2022-0288). Informed consent was obtained from all patients participating in the study. For a period of 2 years and 6 months, between January, 2016 and June, 2018, the data of 519 patients who were enrolled at the General Hospital of Ningxia Medical University and had undergone radical surgery for stomach cancer were examined. Radiation therapy for stomach cancer was performed on all patients, involving distal, proximal and total gastrectomy. The TNM staging criteria were determined according to the eighth edition of the diagnostic criteria for gastric cancer, which was jointly formulated by the American Cancer Consortium (AJCC) and the Union for International Cancer Control (UICC) in 2016, and the clinical staging of the patients in the present study was determined as stage I-IV (M0).

### Inclusion and exclusion criteria

The inclusion criteria were as follows: Patients who had undergone a gastroscopic pathologic examination for gastric cancer and had received successful radical surgery for stomach cancer; patients with complete clinicopathologic data; and those with follow-up data. The exclusion criteria were the following: Individuals who had emergency surgery to treat a stomach cancer-related perforation and obstruction; those who were found to have distant metastasis or abdominal peritoneal metastasis before and during the surgery; those who were combined with other tumors; those who succumbed after the surgery due to complications; those who were lost to follow-up; and those who had incomplete data.

### Gastric cancer specimens and pathological examination

Distal gastrectomy, proximal gastrectomy and total gastrectomy were performed in 348 (67.1 %), 67 (13.9%), and 104 (20.0%) of the specimens obtained, respectively. The isolated specimens were fixed in a 10% neutral formalin solution for 24 hours at room temperature (25 °C) and the fixed tissues were dehydrated and macerated, and representative sections were prepared into paraffin-embedded specimens. IHC was performed using primary antibody for p53 (clone DO-7, cat. no. ZM0408, 1:200 dilution; Zhongshan GoldenBridge Biotechnology, Beijing, China) and BOND-III autostainer (Leica Biosystems Nussloch GmbH, Nussloch, Germany). Immunohistochemical sections were prepared after sectioning, and the microscopic features of the tissues were recorded by two diagnostic pathologists under microscopic observation (OLYMPUS-BX51, OLLYMPU, Tokyo, Japan). Patients received adjuvant therapy according to the recommendations^18^. It was customary to advise patients with advanced GC to undergo 6-8 cycles of adjuvant chemotherapy based on 5-fluorouracil (5-FU) [Oxaliplatin plus Capecitabine or S-1 (XELOX/SOX)] following surgery every 3 weeks.

### Data collection and follow-up

The data of the patient, including sex, age, carcinoembryonic antigen (CEA), carbohydrate antigen (CA)19-9, tumor location, tumor dimensions, invasion depth, metastasis to lymph nodes, lymphovascular and perineural invasions, differentiation and Lauren classification, as well as the p53 expression pattern in gastric cancer tissues, chemotherapy, and the pattern of recurrence and metastasis were analyzed (the pattern of tumor recurrence included local recurrence, peritoneal metastasis and distant metastasis). The 5-year overall survival (OS) rate and recurrence-free survival (RFS) rate were also recorded. Patient survival and recurrence status information were recorded using outpatient review or telephone follow-up. Following surgery, the patients underwent physical examinations, imaging tests (thoracic, abdominal, pelvic CT scan, magnetic resonance imaging and laboratory testing (CEA and CA19-9). A gastroscopy was performed every 3 months for the first 2 years, and subsequently every 6 months for the following 3 to 5 years, followed by once a year after that. The follow-up time was from the month after the patient's surgical procedure until mortality or until follow-up, with at least 5 years as the observation time point. The follow-up was as of September 30, 2023. OS was defined as the amount of time that passed between surgery and the last follow-up or mortality from any cause. The time between surgery and a recurrence or the final follow-up was termed RFS (time to mortality from any cause).

### IHC and interpretation

For the IHC of p53, an antibody to p53 was applied, and automated staining was performed according to the manufacturer's protocol. A semi-quantitative ternary classifier was used to detect p53 expression, and two pathologists collaborated to decide on the reporting method. In addition, two diagnostic pathologists in consultation reported the patterns of p53 expression. A total of three patterns were observed: i) Heterogeneous (wild-type), with nuclear staining that differed in intensity and percentage; ii) overexpression type, with strong staining that spread across >90% of tumor cells; and iii) absence, with no staining in >90% of tumor cells 16,17.

### Statistical analysis

The primary objective of the present study was to identify statistically significant associations between p53 mutant expression patterns and different factors, mainly to determine how the p53 mutant pattern affects RFS and OS. Fisher's exact test and Pearson's Chi-squared (χ2) test were used to evaluate the categorical variables, and propensity score matching were used for the statistical analyses. The 5-year RFS and OS rates of patients with both the p53 mutant and wild-type patterns were determined using Kaplan-Meier analysis and Cox regression tests. Patients with all forms of recurrence (locoregional, peritoneal, or distant), including those with multiple recurrences, were included in the recurrence group when investigating risk variables for first recurrence sites, while the remaining patients comprised the control group. Cox proportional risk models were used for both univariate and multivariate analyses in order to identify the determinants of survival and recurrence. To exclude selection bias among the patients before comparisons, confounding factors were adjusted between patients with p53 wild-type and patients with p53 mutant pattern using 1:1 propensity score matching techniques in SPSS 26.The caliper width was 0.2. A value of P<0.05 was considered to indicate a statistically significant difference. The statistical software SPSS (version 26.0; IBM Corp.) was used for all analyses.

## Results

### Basic characteristics of the patients and p53 expression patterns

**Figure 1:**
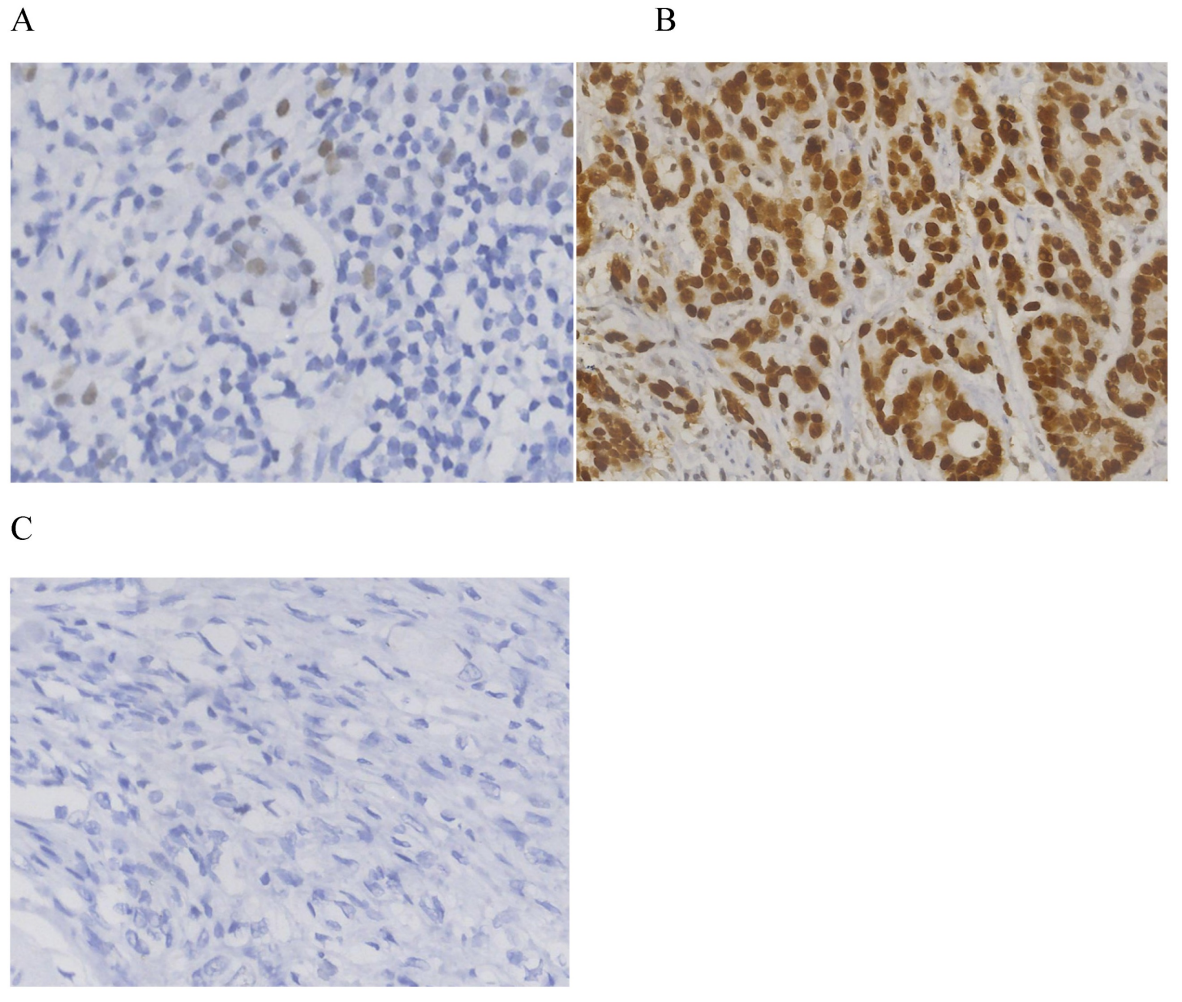
Immunohistochemistry for p53. (A) Heterogeneous nuclear positivity with variable staining intensity was regarded as wild-type. (B) Diffuse positivity >90% with strong nuclear staining (B) and rarely stained tumor cells (<10%) (C) were regarded as overexpression and absence patterns, respectively.

**Figure 2:**
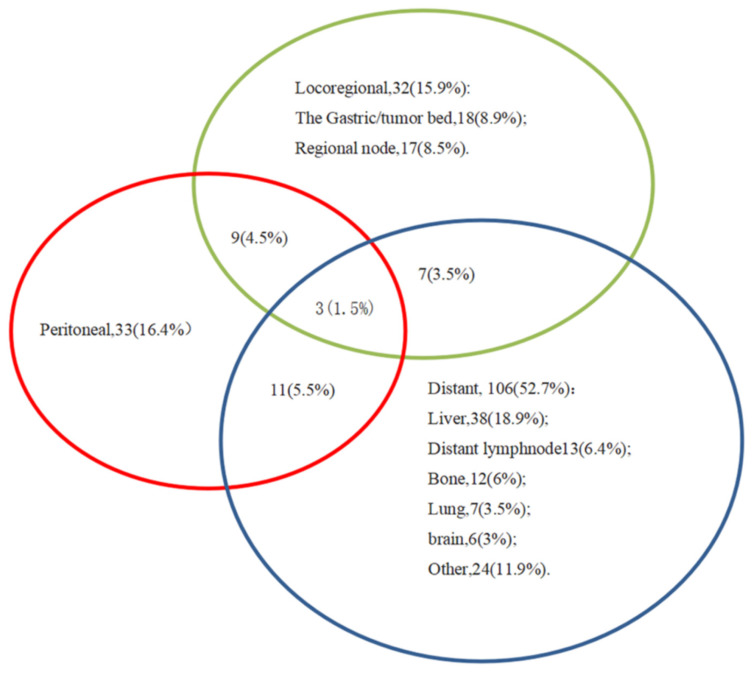
Venn diagram illustrating the recurrence patterns in 201 patients.

**Figure 3:**
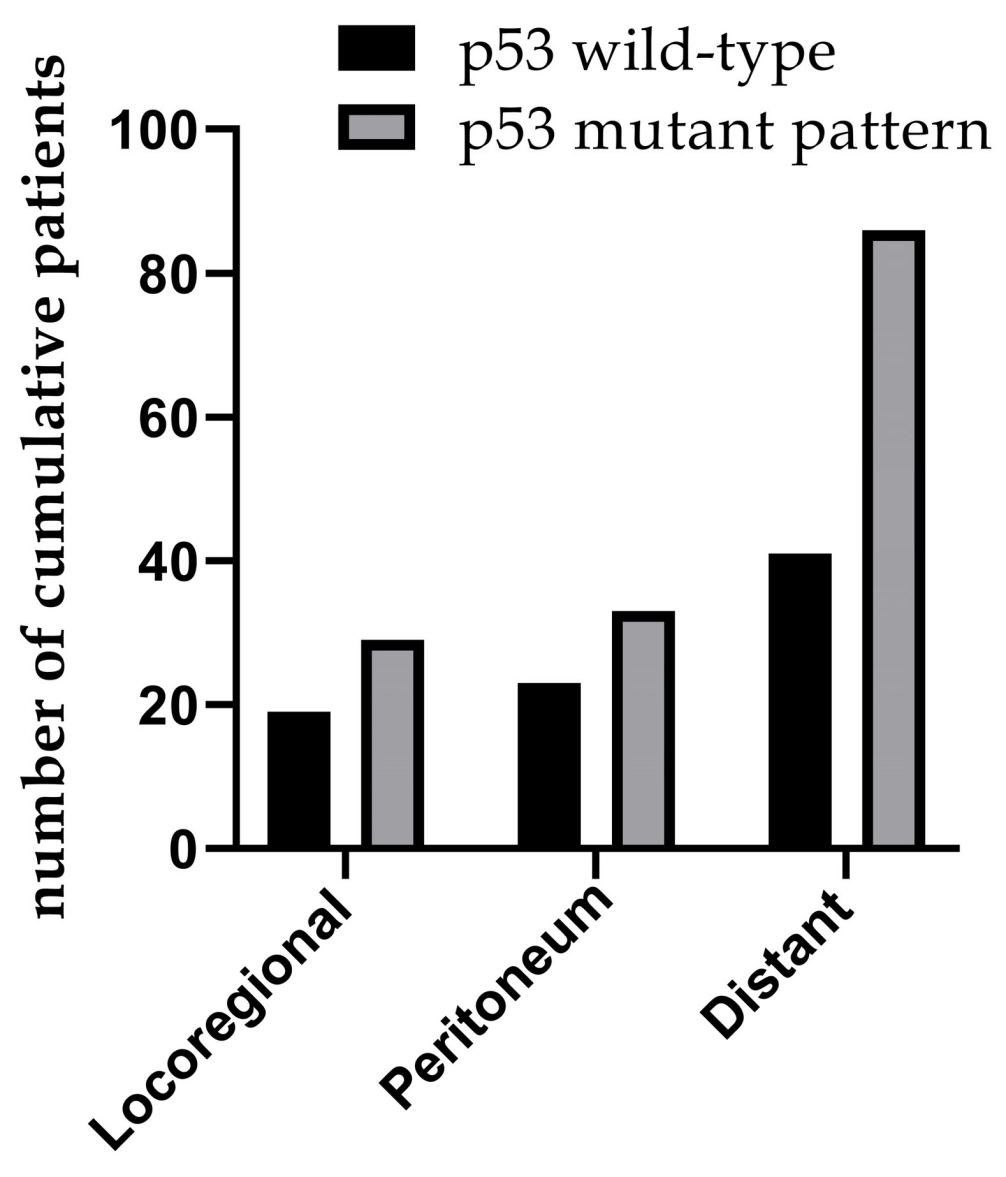
Recurrence patterns involving both p53 mutant and wild-type patterns.

The clinicopathological data of 576 patients who underwent radical surgery for stomach cancer were analyzed in the present study, and complete data were available for 519 patients (90%) in total. There were 117 (22.5%) females and 402 (77.5%) males. The median age was 60, with ages ranging from 23 to 86 years. In total, three types of p53 staining patterns were identified: Wild-type, as heterogeneous nuclear positivity with variable staining intensity (Fig. 1A); >90% of diffuse positivity with strong nuclear staining as the overexpression type (Fig.1B); and rare staining, with <10% of tumor cells being stained; this was regarded as an absence pattern (Fig. 1C). Of the patient tissues, 51.6% (268/519) exhibited the p53 wild-type pattern and 48.4% (251/519) exhibited the p53 mutant pattern, with 97 (18.9%) exhibited the overexpression pattern and 154 (29.7%) the absence pattern. During the time of follow-up, 259 patients (49.9%) succumbed due to gastric cancer and 201 patients (38.7%) developed recurrence. In addition, local recurrence, peritoneal metastasis and distant metastasis occurred in 45 patients (8.7%), 56 patients (10.8%) and 128 patients (24.7%), respectively (Figs. 2 and 3) For the classification of the wild-type and mutant pattern of the p53 group, differences were observed among the CEA levels, pT stage and distant metastasis (Table 1). A comparison of the clinical features between the wild-type and mutant pattern based on the OS and clinicopathological characteristics of the patients and the p53 expression pattern revealed that there was a statistically significant difference between the wild-type and mutant patterns of p53 expression, and pT stage and distant metastasis (P<0.001; Table 2). As can be seen by the bar graph, it was found that among all metastatic patterns, distant metastasis was more common in the p53 mutant pattern, and there was not much difference in local recurrence or peritoneal metastasis (Fig. 3).

### OS and RFS analysis stratified by the p53 staining pattern

**Figure 4:**
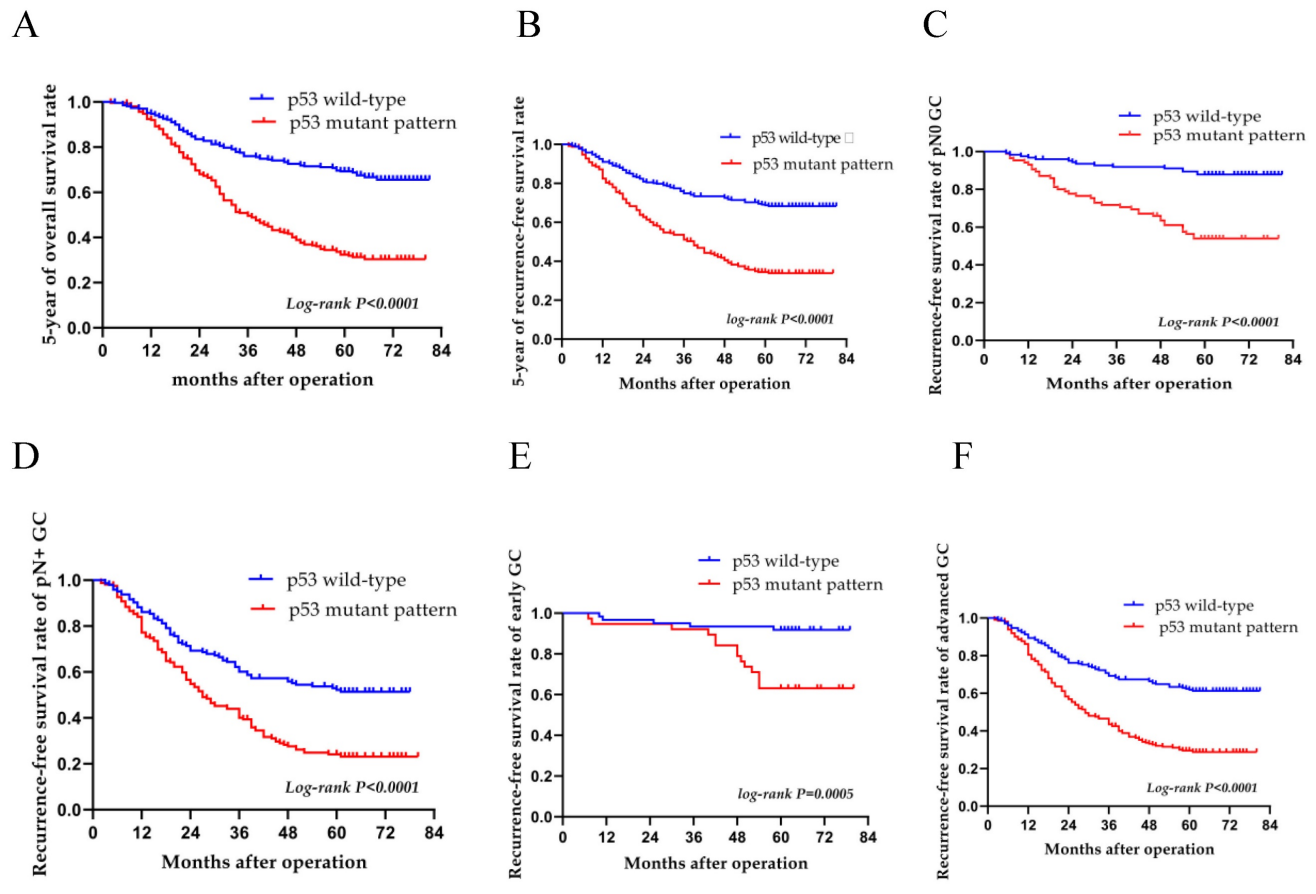
Overall survival and recurrence-free survival of patients with both the p53 wild-type and mutant patterns. Kaplan-Meier curves for (A) overall survival and (B) recurrence-free survival over a period of 5 years with the p53 wild-type and p53 mutant pattern. Patients with the p53 mutant pattern had low (A) overall survival and (B) recurrence-free survival rates in all patients. In subgroup analysis, the recurrence-free survival rate was lower in patients with the p53 mutant pattern than in those with the wild-type pattern as regards both (C) pN0 or (D) pN+, and (E) early and (F) advanced-stage gastric cancer.

A median follow-up period of 47.1 months was observed for patients over a follow-up period of 2-81 months; the RFS rate was 50.1% and the total 5-year survival rate was 50.7%. The 5-year OS rates of the patients with the p53 wild-type and p53 mutant pattern were 68.7 and 31.9%, respectively (log-rank, P<0.0001) (Fig. 4A). The 5-year RFS variants of patients with the p53 wild-type and p53 mutant pattern were 67.5 and 31.5%, respectively (log-rank, P<0.0001) (Fig. 4B). Compared to wild-type patients, those with the p53 mutant pattern had worse OS and RFS rates (P<0.001 for both). In subgroup analyses, there was a significant difference between the wild-type and mutant pattern (P<0.001) in terms of distant metastasis (Table 3); there was a significant difference between the p53 wild-type and p53 mutant pattern (P<0.001) in pN0 and pN+ gastric carcinomas (P<0.001 and P=0.014, respectively). The levels of CEA and CA19-9 also differed significantly in pN0 gastric carcinomas. For the pN0 and pN+ groups, the 5-year OS rate was 71.7 and 36.2%, and the 5-year RFS rate was 71.2 and 35.2%, respectively (P<0.001) (Table 4, and Fig. 4C and D). The p53 mutant pattern exhibited a significant ability in determining the RFS of patients with advanced-stage gastric cancer (P<0.0001). Multivariate analysis revealed that the p53 mutant pattern, a more advanced pN stage, and a more advanced pT stage were all strongly associated with disease recurrence (P<0.05) (Table 5, and Fig. 4E and F).

### p53 mutant pattern predicts distant metastasis

In the Cox univariate regression analysis, the p53 mutant pattern was a significant predictor of distant metastasis [relative risk (RR)=2.881, P<0.001) and OS (RR=2.809, P<0.001), particularly in advanced-stage gastric cancer. In the Cox multivariate regression analysis, the pN staging and p53 staining pattern were significant variables (both P<0.001) and the p53 mutant staining pattern remained a key predictor of distant metastasis following gastric cancer surgery (RR=2.767, P< 0.001) (Table 3 and V). Furthermore, age, pT stage, pN stage, local recurrence, peritoneal metastasis, distant metastasis and the p53 mutant pattern were independent prognostic variables that affected the survival of patients with gastric cancer post-operatively (P<0.05). By contrast, other independent prognostic factors, such as CEA, CA19-9, Lauren classification, differentiation, lymphovascular invasion, and perineural invasion did not yield statistically significant results (Table 2).

### The p53 mutant pattern predicts OS and distant metastasis after propensity score matching

After propensity score matching, 189 patients with a p53 wild-type and 189 patients with a p53 mutant pattern were extracted for analysis, and the major clinical factors were nearly equivalent between the two groups (n=189 in each) (Table 6). The 5-year overall survival rate in patients with the p53 mutant pattern (n = 189) was worse than that in the patients with p53 wild-type (n = 189) and with significant differences (log-rank P<0.01) (Table 7). The study was statistically significant after Cox univariate and multivariate regression analysis, which revealed that the mutant pattern of p53 is an independent prognostic factor impacting distant metastases following curative gastrectomy for advanced-stage gastric cancer (p = 0.48) (Table 8).

## Discussion

The present study demonstrated that the mutant pattern of p53 determined using IHC can be used as a key prognostic factor for the OS and distant metastasis of patients with gastric cancer. p53 may help to better respond to tumor characteristics and may thus be used to accurately determine clinical prognosis. In the present study, the p53 mutant pattern was found in 48.4% of patients with gastric cancer, and the results revealed that the p53 mutant pattern was more aggressive than the wild-type. It was found that the 5-year OS rate of patients with gastric cancer was 68.7% for patients classified as p53 wild-type, and 31.9% for those with the p53 mutant pattern, and there was also a significant difference in the 5-year disease-free survival rate between the two groups. In gastric cancer, the 5-year RFS rate was 67.5% for patients classified as p53 wild-type, and 31.5% for those with the p53 mutant pattern. Thus, p53 IHC staining assays can help to determine the likelihood of survival and may also assist in selecting the optimal treatment strategy for patients with gastric cancer. The p53 mutant pattern may cause the tumor to return and may alter the patient's chance of survival due to poor biological oncological properties, such as promoting rapid cancer growth and negatively affecting the response to chemo-radiation therapy.

One of the main findings of the present study was that distant metastasis was the most frequent among all metastasis types, which is in accordance with the findings of the study by Tang *et al.* 5. Further findings revealed that mutant p53 was closely associated with distant metastasis in advanced stages of gastric cancer. The present study collected 519 surgical specimens and examined survival and metastasis, both on the patients overall and by subgroup analyses, including pN0 and pN+, and early and advanced stages of gastric cancer. Through multivariate regression analyses, an association was found between distant metastasis and both lymph node metastasis and the p53 mutant pattern. Significant differences were found between patients with lymph node metastases and those without metastases, as well as between patients with the p53 mutant pattern and those with the wild-type. Metastasis to the lymph nodes was a poor factor for distant metastasis in the study by Tang *et al.* 5, as well as in another study 3; this is consistent with the findings of the present study. Previous studies 16,17 have also found that the recurrence pattern of gastric cancer in patients without lymph node metastasis differs from pT staging: Patients with pT1~2 illness have been found to have the highest frequency of local recurrence (57.1%), followed by patients with pT3 disease (57.1%) and pT4a disease (66.7%) in terms of distant recurrence.

Patients with pT1-associated disease have been found to have the highest frequency of locoregional recurrence (57.1%), followed by those with pT3 disease (57.1%) and pT4a disease (66.7%) in terms of distant recurrence. Following a curative gastrectomy for gastric cancer, CEA is probably helpful in detecting recurrence 19. In the present study, it was also found that pT and CEA were associated with the p53 mutant pattern and were thus involved in gastric cancer recurrence and metastasis. Following a curative gastrectomy of all the patients, the condition of the lymph nodes is another key factor related to distant metastases 5. In the present study, in subgroup analyses, categorizing pN0 and pN+ and those with or without lymph node metastasis, the p53 mutant pattern was closely associated with distant metastasis. The present study also examined the pN0 and pN+ medical history records to determine whether lymph node metastasis with the p53 mutant pattern was a key factor (P=0.007 and P<0.001). Furthermore, when comparing the early and progressive stages, it was discovered that patients with progressive gastric cancer with the p53 mutant pattern had a higher distant metastasis rate and a lower RFS rate. It was also found that the p53 mutant pattern was more frequent than the wild-type in patients with gastric cancer local recurrence and peritoneal metastasis. The p53 mutant pattern is ~2-fold more common than the wild-type, and is observed in a larger proportion of distant metastasis cases; in terms of OS, the p53 mutant pattern is significantly less frequent than the wild-type. It is suggested that the p53 mutant pattern is indicative of a poor survival rate and a higher recurrence rate. This indicated that in patients with progressive gastric cancer, distant metastasis was significantly more common among those harboring the p53 mutant pattern than the p53 wild-type pattern (P<0.001). Therefore, a simple p53 IHC analysis can be used to effectively predict recurrence and metastasis regardless of the molecular subtype. Studies have also been conducted on distant metastasis in different cancers. According to Huang *et al.* 20, colon cancer patients with p53 mutations had a higher risk of distant metastasis (OR 1.35, 95% CI 1.06-1.72). Van Egeren D *et al.* 21 have also documented a significant correlation between TP53 gene alterations and new distant metastases (HR = 1.43,95% CI 1.09-2.90) in non-small cell lung cancer in 759 patients with stage I-III disease. It implied that distant metastases are common in malignancies and should be thoroughly investigated. In this study, the 5-year overall survival rate in patients with p53 mutant pattern (n=189) was worse than that in the patients with p53 wild-type after the propensity score matching; the mutant pattern of p53 is an independent prognostic factor impacting distant metastases following curative gastrectomy for advanced-stage gastric cancer. This result is consistent with studies before propensity score matching. The conclusion that the p53 mutant pattern is a reliable indicator of distant metastases following a curative gastrectomy for advanced-stage gastric cancer validates the findings before and after matching on the propensity score.

Numerous academics have previously investigated the pattern of recurrence following radical gastrectomy for cancer 22-25. However, the results obtained vary due to the variability of the study populations. Therefore, the analysis of the association of recurrence patterns with clinicopathological data has currently become a hot research topic. Kim *et al.* examined a group of patients who had recurrent gastric cancer and discovered that more than half of those with stage I gastric cancer had metastases in other organs. In patients with stage III gastric cancer, peritoneal metastases were the most common type. Toriumi *et al.* examined 253 cases out of 1,204 individuals for a European journal analysis of the JCOG1001 trial and found that hematogenous recurrence (distant metastasis) was the most common type. This was followed by peritoneal metastasis and then local recurrence. In addition, Akcay *et al.* predicted distant metastasis as the most common type using a machine learning model. Prior research indicates that tumor cells spread throughout the body via lymph node vascular cutlets 26. Therefore, the present study conducted an in-depth analysis and found an association between distant metastasis and the mutant p53 status.

Currently, the IHC staining of p53 is the most common method used to assess the mutant p53 status. There are more research studies on the relationship between IHC expression of p53 and p53 mutant status 27-31. However, due to the different interpretations of p53 IHC, it has not been confirmed in numerous types of cancer, including nodal gastric cancer. In a previous study 29 on endometrial cancer, p53 expression was categorized into wild-type, overexpression and complete deletion. There have been prior reports linking the overexpression of p53 to a poor prognosis or disease progression in patients with colorectal cancer 32. Recently, there have been several attempts to create IHC reading methods for p53 and mutant p53 that can be effectively used in stomach cancer, with the use of p53 expression as a predictor. In a study from Central Europe, Schoop *et al.* used their own IHC evaluation algorithms to examine the status of mutant p53 in IHC. Different p53 IHC evaluation algorithms, on the other hand, could not be used to determine whether a case of CIN gastric cancer would have mutant p53 33. According to the study by Hwang *et al.* 34 on endometrial and ovarian cancer 28, the level of p53 expression was divided into three groups: Strong expression (>10% of tumor cells had strong positivity), expression deficiency (tumor cells did not have nuclear staining) and weak expression (weak positivity, scattered or patchy positivity).In this study, the simple yin and yang dichotomy of p53 was discarded, and the triple classification method of many scholars 28-31,34,35 was adopted to classify p53 staining in the tissues of patients after radical gastric cancer surgery. Meanwhile, this study provided a comprehensive analysis of the metastatic recurrence of gastric cancer after radical gastric cancer surgery, including local recurrence, peritoneal metastasis, and distant metastasis, whereas previous studies by Korean scholars 34,35 only included two types of local recurrence and distant metastasis; furthermore, the population included in this study included patients with early and progressive gastric cancer, whereas the population included in previous studies included patients with gastroscopic mucosal resection, and the patients with gastroscopic mucosal resection were in the early stages, and there was a selective bias. Therefore, the present study revealed the value of p53 mutant patterns in gastric cancer and used it as a clinical guide.

In a previous Science Translational Medicine study 36, it was found that mRNA therapy was potentially effective for patients with p53 mutant cancer. Shi Jinjun *et al.* at Harvard Medical School used nanoparticles to introduce mRNA for p53 into p53-deficient tumor cells, and it inhibited the growth of tumor cells 37. This approach was used to obtain significant antitumor effects when combined with the mammalian target of rapamycin inhibitor, everolimus 36. Currently, research on treatment options for patients with HER-2-positive advanced-stage gastric cancer is stagnant, and studies on new combination chemotherapeutic regimens have failed to further prolong the OS of patients 38. The search for new targets is a key direction in research, and p53 warrants further in-depth analyses. Patients with p53 overexpression have a significantly lower OS rate, indicating that the TP53 mutation is linked to a worse prognosis due to metastasis, increased chemotherapeutic resistance and tumor growth. Numerous substances with the ability to disrupt or reactivate mutant p53 are currently under investigation. Among these, clinical trial approvals have been granted for APR-246, COTI-2, SAHA and PEITC 39. To date, APR-246 is the most prominent drug that has entered phase III clinical trials 40, which requires further in-depth research; thus, p53 should be given due attention.

There are several limitations to the present study which should be mentioned. A further multicenter investigation is required as the present study was a single-center retrospective study with a small sample size and possible selective bias. Consequently, in order to validate this prediction model, a multicenter investigation needs to be performed with a larger sample size. Second, the present study only used IHC for the molecular assessment of p53 mutations, and the overexpression of p53 in IHC was used as a proxy for TP53 mutations, which should be validated by the addition of next-generation sequencing comparisons. The authors aim to continue to gather information from patients with early-stage gastric cancer in order to assess the impact of the p53 mutant pattern on the prognosis and recurrence of early-stage gastric cancer. This is due to the fact that the recurrence rate of patients with early-stage gastric cancer is lower and the difference in recurrence between patients with p53 mutant and wild-type pattern is not statistically significant.

In conclusion, the present study demonstrated that the p53 mutant staining pattern of patients with advanced-stage gastric cancer demonstrated a poor overall survival and a high distant metastasis rate compared with the wild-type pattern; this suggests that a p53 mutant status identified using IHC in the tissues of patients with gastric cancer following radical resection is a key predictor of prognosis or recurrence. In view of the fact that p53 is easy to analyze using IHC in gastric cancer specimens, which is helpful for surgery, adjuvant therapy and follow-up, it is suggested that p53 IHC staining stratification method should be routinely performed in clinical practice. This would guide the clinical emulation of patients and would provide the basis for the precise assessment of the long-term survival and prognosis of patients following radical resection for stomach cancer. In addition, it would also aid the individualized treatment of patients with gastric cancer, with good prospects for clinical application.

## Figures and Tables

**Table 1:** Clinical characteristics of the patients with p53 staining patterns.

Characteristics	Total cases N (%)	wild-type (N =268) N (%)	mutant pattern (n =251) N (%)	P-value
Gender				0.883
Male	402(77.5)	207(77.2)	195(77.6)	
Female	117(22.5)	61(22.8)	56(22.4)	
Age, years				0.220
<60	246(47.4)	134(50)	112(44.7)	
≥60	273(52.6)	134(50)	139(55.3)	
CEA (ng/ml)				0.025
≥5	103	43(16.0)	60(23.9)	
<5	416	225(84.0)	191(76.1)	
CA199(ng/ml)				0.065
≥37	74(14.3)	31(11.6)	43(17.1)	
<37	445(85.7)	237(88.4)	208(82.9)	
Location				0.220
Upper	115(22.2)	51(19.0)	64(25.5)	
Middle	300(57.8)	164(61.2)	136(54.2)	
Lower	104(20.0)	53(19.8)	51(20.3)	
Size (cm)				0.12
≥3	368(70.9)	182(67.9)	186(74.1)	
<3	151(29.1)	86(32.1)	65(25.9)	
pT stage				0.011
T1	100(19.3)	61(22.8)	39(15.5)	
T2	91(17.5)	56(20.9)	35(13.9)	
T3	43(8.3)	20(7.5)	23(9.2)	
T4	285(54.9)	131(48.8)	154(61.4)	
pN stage				0.070
N0	212(40.8)	124(46.3)	88(35.1)	
N1	96(18.5)	46(17.1)	50(19.9)	
N2	80(15.4)	39(14.5)	41(16.3)	
N3	131(25.3)	59(48.8)	72(28.7)	
Vascular invasion				0.392
Yes	267(51.4)	133(49.6)	134(53.4)	
No	252(48.6)	135(50.4)	117(46.6)	
Perineural invasion				0.308
Yes	230(44.3)	113(42.2)	117(46.6)	
No	289(55.7)	155(57.8)	134(53.4)	
Differentiation				0.062
Well/Moderate	122(23.5)	72(26.9)	50(19.9)	
Poor/signet ring cell	397(76.5)	196(73.1)	201(80.1)	
Lauren classification				0.999
Intestinal	141(27.2)	73(27.2)	68(27.1)	
Diffuse	184(35.5)	95(35.4)	89(35.5)	
Mixed	194(37.3)	100(37.4)	94(37.4)	
Locoregional recurrence				0.160
Yes	45(8.7)	19(7.1)	26(10.4)	
No	474(91.3)	249(92.9)	225(89.6)	
Peritoneal recurrence				0.078
Yes	56(10.8)	24(8.9)	32(12.7)	
No	463(89.2)	244(90.1)	219(87.3)	
Distant metastasis				0.001
Yes	128(27.7)	41(15.3)	87(34.7)	
No	391(75.3)	227(84.7)	164(65.3)	

**Abbreviation CEA:** carcinoembryonic antigen**; CA199:** Carbohydrate antigen 199.

**Table 2:** Univariate and multivariate Cox regression analysis of patients for overall survival.

Characteristics	Univariate analysis			Multivariate analysis	
	HR (95% CI)	P-value		HR (95% CI)	P-value
Gender (male vs female)	0.956(0.716-1.276)	0.760			
Age (≥60yrs vs <60yrs)	1.811(1.406-2.333)	<0.001		1.562(1.196-2.040)	0.001
CEA (≥5ng/m vs <5ng/ml)	0.681(0.512-0.906)	0.008		0.862(0.633-1.174)	0.347
CA199 (≥27ng/m vs <27ng/ml)	1.740(1.271-2.382)	0.001		1.161(0.835-1.615)	0.374
Location					
Upper	Reference	0.098		Reference	0.893
Middle (upper vs middle)	0.737(0.551-0.985)	0.039		1.074(0.782-1.475)	0.660
Lower (upper vs lower)	0.735(0.509-1.060)	0.100		1.088(0.726-1.629)	0.684
Size (≥3cm vs <3cm)	2.481(1.803-3.415)	<0.001		1.365(0.937-1.988)	0.105
pT (yes vs no)					
T1	Reference	<0.001		Reference	0.045
T2 (T2 vs T1)	2.225((1.261-3.926)	0.006		2.169(1.147-4.102)	0.017
T3 (T3 vs T1)	3.759(2.033-6.950)	<0.001		1.171(0.798-3.693)	0.167
T4 (T4 vs T1)	5.165(3.281-8.290)	<0.001		2.307(1.204-4.423)	0.012
pN					
N0	Reference	<0.001		Reference	0.009
N1 (N1 vsN0)	2.542(1.747-3.699)	<0.001		1.858(1.224-2.821)	0.004
N2 (N2 vsN0)	3.685(2.549-5.327)	<0.001		1.755(1.142-2.699)	0.010
N3 (N3 vsN0)	3.946(2.837-5.849)	<0.001		1.975(1.292-3.021)	0.002
Vascular invasion (yes vs no)	1.958(1.522-2.520)	<0.001		0.914(0.669-1.248)	0.571
Perineural invasion (yes vs no)	1.601(1.255-2.044)	<0.001		0.945(0.704-1.268)	0.7041
Differentiated type (yes vs no)	2.039(1.460-2.848)	<0.001		0.757(0.498-1.149)	0.190
Lauren classification					
Intestinal	Reference	<0.001		Reference	0.449
Diffuse (diffuse vs intestinal)	1.669(1.182-2.356)	0.004		1.165(0.779-1.724)	0.457
Mixed (mixed vs intestinal)	2.2(1.574-3.076)	<0.001		1.296(0.858-1.958)	0.217
p53 pattern (wild-type vs mutant pattern)	2.809(2.167-3.642)	<0.001		1.900(1.442-2.505)	<0.001
Locoregional recurrence (yes vs no)	2.481(1.783-3.451)	<0.001		1.528(1.054-2.216)	0.025
Peritoneal recurrence (yes vs no)	3.572(2.622-4.865)	<0.001		1.843(1.306-2.602)	0.001
Distant metastasis (yes vs no)	5.095(3.951-6.570)	<0.001		3.511(2.633-4.682)	<0.001
Postoperative chemotherapy (yes vs no)	1.735(1.287-2.337)	<0.001		0.714(0.508-1.003)	0.052

**Abbreviation CEA:** carcinoembryonic antigen**; CA199:** Carbohydrate antigen 199.

**Table 3:** Cox regression analysis for distant metastasis

Clinical characteristics	Univariate analysis			Multivariate analysis	
	HR (95% CI)	P-value		HR (95% CI)	P-value
Gender (male vs female)	1.0656(0.707-1.604)	0.766			
Age (≥60 yrs vs <60 yrs)	1.136(0.801-1.612)	0.473			
CEA (≥5ng/m vs <5ng/ml)	1.912(1.307-2.797)	0.001		1.286(0.860-1.925)	0.221
CA199 (≥27ng/m vs <27ng/ml)	1.902(1.235-2.928)	0.004		1.321(0.839-2.080)	0.229
Location					
Upper	Reference	0.226			
Middle (upper vs middle)	0.721(0.478-1.087)	0.118			
Lower (upper vs lower)	0.735(0.509-1.060)	0.150			
Tumor size (≥3cm vs <3cm)	2.465(1.555-2.906)	<0.001		1.107(0.658-1.826)	0.702
pT (yes vs no)					
T1	Reference	<0.001		Reference	0.158
T2 (T2 vs T1)	4.467((1.470-13.575)	0.008		2.032(0.611-6.759)	0.248
T3 (T3 vs T1)	7.745(2.427-24.720)	0.001		1.499(0.390-5.766)	0.556
T4 (T4 vs T1)	12.499(4.595-34.001)	<0.001		2.625(0.796-8.650)	0.113
pN					
N0	Reference	<0.001		Reference	<0.001
N1 (N1 vsN0)	2.027((1.062-3.868)	0.32		0.937(0.476-1.846)	0.851
N2 (N2 vsN0)	5.938(3.473-10.152)	<0.001		2.791(1.540-5.059)	0.001
N3 (N3 vsN0)	5.913(3.588-9.744)	<0.001		2.184(1.213-3.933)	0.009
Vascular invasion (yes vs no)	2.818(1.920-4.136)	<0.001		1.249(0.804-1.941)	0.323
Perineural invasion (yes vs no)	1.922(1.352-2.733)	<0.001		0.937(0.654-1.449)	0.894
Differentiated type (yes vs no)	4.024(2.167-7.474)	<0.001		1.417(0.713-2.817)	0.319
Lauren classification					
Intestinal	Reference	<0.001		Reference	0.192
Diffuse (diffuse vs intestinal)	2.688(1.530-4.720)	0.001		1.699(0.908-3.181)	0.098
Mixed (mixed vs intestinal)	3.493(2.012-6.065)	<0.001		1.769(0.943-3.318)	0.217
p53 pattern (wild-type vs mutant pattern)	2.881(1.981-4.189)	<0.001		2.767(1.886-4.058)	<0.001
Locoregional recurrence	0.958(0.501-1.832)	0.897			
Peritoneal recurrence	1.618(0.923-2.834)	0.093			
Adjuvant chemotherapy	3.230(1.912-5.458)	<0.001		1.565(0.901-2.720)	0.112

**Abbreviation CEA:** carcinoembryonic antigen**; CA199:** Carbohydrate antigen 199. HR: Hazard ratio; CI: Confidence interval

**Table 4:** Comparison of patient characteristics between pN0 and pN+ gastric cancer in p53 wild-type and mutant pattern.

Characteristics	PN0 gastric cancer (n=212)					PN+gastric cancer (n=307)			
	Total cases N (%)	wild-type N (%)	mutant pattern N (%)	P-value		Total cases N (%)	wild-type N (%)	mutant pattern N (%)	P-value
Gender				0.230					0.562
Male	170(80.2)	96(77.4)	74(84.1)			232(75.6)	111(77.1)	121(74.2)	
Female	42(19.8)	28(22.6)	14(15.9)			75(24.4)	33(22.9)	42(25.8)	
Age (yrs)									
<60	105(49.5)	60(48.4)	47(53.4)	0.471		141(46.0)	70(48.6)	71(43.6)	0.375
≥60	107(50.5)	64(51.6)	41(46.6)			166(54.0)	74(51.4	92(56.4)	
CEA (ng/ml)				0.008					0.576
≥5	26(12.3)	9(7.3)	17(19.3)			77(25.1)	34(23.6)	43(26.4)	
<5	186(87.7)	115(92.7)	71(80.7)			230(74.9)	110(76.4)	120(73.6)	
CA199 (ng/ml)				<0.001					0.627
≥27	15(7.1)	5(4.0)	10(11.4)			59(19.2)	26(18.1)	33(20.0)	
<27	197(92.9)	119(96.0)	78(89.6)			248(80.8)	118(81.9)	130(80.0)	
Location				0.535					0.452
Upper	34(16.0)	17(13.7)	17(19.3)			81(26.4)	34(23.6)	47(28.8)	
Middle	127(59.9)	77(62.1)	50(56.9)			173(56.4)	86(59.7)	87(53.4)	
Lower	51(24.1)	30(24.2)	21(23.9)			53(17.2)	24(16.7)	29(17.8)	
Tumor size (cm)				0.545					0.762
≥3cm	104(49.1)	63(50.8)	41(46.6)			47(15.3)	23(16.0)	24(14.7)	
<3cm	108(50.9)	61(49.2)	47(53.4)			260(84.7)	121(84.0)	139(85.3)	
pT				0.254					0.514
T1	92(43.4)	57(46.0)	35(39.8)			8(2.6)	4(2.8)	4(2.4)	
T2	53(25.0)	34(27.4)	19(21.6)			38(12.4)	22(15.3)	16 (9.8%)	
T3	10(4.7)	6(4.8)	4(4.5)			33(10.7)	14(9.7)	19(11.7)	
T4	57(26.9)	27(21.8)	30(34.1)			228(74.3)	104(72.2)	124(76.1)	
Lymphovascular invasion				0.551					0.356
Yes	51(24.1)	28(22.6)	23(26.1)			216(70.4)	105(73.0)	111(68.1)	
No	161(75.9)	96(77.4)	65(73.9)			91(29.6)	39(27.0)	52(31.9)	
Perineural invasion				0.132					
Yes	47(22.2)	23(18.5)	24(27.3)			216(70.4)	124(86.1)	93(57.1)	0.016
No	165(77.8)	101(81.5)	64(72.7)			91(29.6)	54(13.9)	70(42.9)	
Differentiation				0.007					0.135
Well/Moderate	90(42.5)	53(42.7)	37(42.0)			32(10.4)	19(13.2)	13(8.0)	
Poor/signet ring cell	122(57.5)	71(57.3)	51(58.0)			275(89.6)	125(86.8)	150(92.0)	
Lauren classification				0.637					0.546
Intestinal	98(46.2)	54(43.5)	44(50.0)			43(14.0)	19(13.2)	24(14.7)	
Diffuse	66(31.1)	40(32.3)	26(29.5)			118(38.4)	55(38.2)	63(38.7)	
Mixed	48(22.7)	30(24.2)	18(20.5)			146(47.6)	70(48.6)	76(46.6)	
Locoregional recurrence				0.319					0.071
Yes	17(8.0)	8(6.5)	9(10.2)			34(11.1)	11(7.6)	23(14.1)	
No	195(92.0)	116(93.5)	79(89.8)			273(88.9)	133(92.4)	140(85.9)	
Peritoneal recurrence				0.382					0.333
Yes	9(4.2)	4(3.3)	5(4.0)			47(15.3)	19(13.2)	28(17.2)	
No	203(95.8)	120(96.7)	83(96.0)			260(84.7)	125(86.8)	135(82.8)	
Distant metastasis				0.007					<0.001
Yes	22(10.4)	7(5.6)	15(17.0)			105(34.2)	34(23.6)	71(43.6)	
No	190(89.6)	117(94.4)	73(83.0)			202(65.8)	110(76.4)	92(56.4)	

**Abbreviation CEA:** carcinoembryonic antigen**; CA199:** Carbohydrate antigen 199.

**Table 5:** Comparison of the characteristics of patients with early- and advanced-stage gastric cancer in association with the p53 wild-type and p53 mutant pattern.

Characteristics	Early gastric cancer (n=100)					Advanced gastric cancer (n=419)			
	Total cases N (%)	wild-type N (%)	mutant pattern N (%)	P-value		Total cases N (%)	wild-type N (%)	mutant pattern N (%)	P-value
Gender				0.688					0.814
Male	74(74.0)	46(75.4)	28(71.7)			91(21.7)	46(22.2)	45(21.2)	
Female	26(260)	15(24.6)	11(28.3)			328(78.3)	161(77.8)	167(78.8)	
Age (yrs)				0.715					0.171
<60	51(51.0)	32(52.4)	19(48.7)			221(52.7)	102(49.3)	119(56.1)	
≥60	49(49.0)	29(47.6)	20(51.3)			198(47.3)	105(50.7)	93(43.9)	
CEA (ng/ml)				0.323					0.106
≥5	5(5.0)	2(3.3)	3(7.7)			98(23.4)	41(19.8)	57(26.9)	
<5	95(95.0)	59(96.7)	36(92.3)			321(76.6)	166(80.2)	155(73.1)	
CA199(ng/ml)				0.318					0.195
≥37	3(3.0)	1(1.6)	2(5.1)			71(16.9)	30(14.5)	41(19.3)	
<37	97(97.0)	60(98.4)	37(94.9)			348(83.1)	177(85.5)	171(80.7)	
Location				0.640					0.163
Upper	17(17.0)	9(14.8)	8(20.5)			98(23.4)	42(20.3)	56(26.4)	
Middle	56(56.0)	34(55.7)	22(56.4)			244(58.2)	130(62.8)	114(53.8)	
Lower	27(27.0)	18(29.5)	9(23.1)			77(18.4)	35(16.9)	42(19.8)	
Tumor size (cm)				0.297					0.214
≥3cm	71(71.0)	41(67.2)	30(76.9)			80(19.1)	45(21.7)	35(16.5)	
<3cm	29(29.0)	20(32.8)	9(23.1)			339(80.9)	162(78.3)	177(83.5)	
pN				0.506					0.105
N0	92(92.0)	57(93.4)	35(89.7)			120(28.6)	67(32.4)	53(25.0)	
N+	8(8.0)	4(6.6)	4(10.3)			299(71.4)	140(37.6)	159(75.0)	
Lymphovascular invasion				0.645					0.840
Yes	96(96.0)	59(96.7)	37(94.9)			156(37.2)	76(36.7)	80(37.8)	
No	4(4.0)	2(3.3)	2(5.1)			263(62.8)	131(63.3)	132(62.2)	
Perineural invasion			1						0.769
Yes	98(98.0)	59(96.7)	39(100)	0.254		191(45.6)	96(46.4)	95(44.8)	
No	2(2.0)	2(3.3)	0(0.0)			228(54.4)	111(53.6)	117(55.2)	
Differentiation				0.955					0.312
Well/Moderate	67(67.0)	41(67.2)	26(66.7)			55(13.1)	31(15.0)	24(11.3)	
Poor/signet ring cell	33(33.0)	20(32.8)	13(33.3)			364(86.9)	176(85.0)	188(88.7)	
Lauren classification				0.759					0.813
Intestinal	63(63.0)	37(60.6)	26(66.7)			78(18.6)	36(17.4)	42(19.8)	
Diffuse	23(23.0)	14(22.9)	9(23.1)			161(38.4)	81(39.1)	80(37.8)	
Mixed	14(14.0)	10(16.5)	4(10.3)			180(43.0)	90(43.5)	90(42.4)	
Locoregional recurrence				0.747					0.068
Yes	2(2.0)	1(1.6)	1(2.6)			49(11.7)	18(8.7)	31(14.6)	
No	98(98.0)	60(98.4)	38(97.4)			370(88.3)	189(91.3)	181(85.4)	
Peritoneal recurrence				0.747					0.191
Yes	2(2.0)	1(1.6)	1(2.6)			54(12.9)	22(10.6)	32(15.1)	
No	98(98.0)	60(98.4)	38(97.4)			365(87.1)	185(89.4)	180(84.9)	
Distant metastasis, n (%)				0.296					<0.001
Yes	4(4.0)	1(1.6)	3(7.7)			123(29.4)	40(19.3)	83(39.2)	
No	96(96.0)	60(98.4)	36(92.3)			296(70.6)	167(80.1)	129(60.8)	

**Abbreviation CEA:** carcinoembryonic antigen**; CA199:** Carbohydrate antigen 199.

**Table 6:** Clinical characteristics of the patients with p53 staining patterns before and after matching on the propensity score.

Characteristics	Before matching			After matching		
	wild-type (N=268) N (%)	mutant pattern (n=251) N (%)	P-value	wild-type (N=189) N (%)	mutant pattern (n=189) N (%)	P-value
Gender			0.883			0.549
Male	207(77.2)	195(77.6)		148	143	
Female	61(22.8)	56(22.4)		41	46	
Age, years			0.220			0.414
<60	134(50)	112(44.7)		89	82	
≥60	134(50)	139(55.3)		100	107	
CEA (ng/ml)			0.025			0.502
≥5	43(16.0)	60(23.9)		32	37	
<5	225(84.0)	191(76.1)		157	152	
CA199 (ng/ml)			0.065			0.603
≥37	31(11.6)	43(17.1)		24	21	
<37	237(88.4)	208(82.9)		165	162	
Location			0.220			0.748
Upper	51(19.0)	64(25.5)		33	41	
Middle	164(61.2)	136(54.2)		116	104	
Lower	53(19.8)	51(20.3)		40	44	
Size (cm)			0.12			0.414
≥3	182(67.9)	186(74.1)		128	133	
<3	86(32.1)	65(25.9)		63	56	
pT stage			0.011			0.177
T1	61(22.8)	39(15.5)		42	35	
T2	56(20.9)	35(13.9)		40	34	
T3	20(7.5)	23(9.2)		13	18	
T4	131(48.8)	154(61.4)		94	102	
pN stage			0.070			0.495
N0	124(46.3)	88(35.1)		88	77	
N1	46(17.1)	50(19.9)		29	40	
N2	39(14.5)	41(16.3)		30	26	
N3	59(48.8)	72(28.7)		42	46	
Vascular invasion			0.392			0.319
Yes	133(49.6)	134(53.4)		89	91	
No	135(50.4)	117(46.6)		100	98	
Perineural invasion			0.308			0.104
Yes	113(42.2)	117(46.6)		71	86	
No	155(57.8)	134(53.4)		118	103	
Differentiation			0.062			0.205
Well/Moderate	72(26.9)	50(19.9)		54	44	
Poor/signet ring cell	196(73.1)	201(80.1)		135	145	
Lauren classification			0.999			0.294
Intestinal	73(27.2)	68(27.1)		60	58	
Diffuse	95(35.4)	89(35.5)		73	62	
Mixed	100(37.4)	94(37.4)		56	69	
Locoregional recurrence			0.160			0.858
Yes	19(7.1)	26(10.4)		19	20	
No	249(92.9)	225(89.6)		170	169	
Peritoneal recurrence			0.078			0.494
Yes	24(8.9)	32(12.7)		17	21	
No	244(90.1)	219(87.3)		172	168	
Distant metastasis			0.001			0.09
Yes	41(15.3)	87(34.7)		41	34	
No	227(84.7)	164(65.3)		148	155	

**Abbreviation CEA:** carcinoembryonic antigen**; CA199:** Carbohydrate antigen 199.

**Table 7:** Univariate and multivariate Cox regression analysis of patients for overall survival after matching on the propensity score(n=378).

Characteristics	Univariate analysis			Multivariate analysis	
	HR (95% CI)	P-value		HR (95% CI)	P-value
Gender (male vs female)	0.854(0.615-1.185)	0.344			
Age (≥60yrs vs <60yrs)	1.922(1.420-2.603)	<0.001		0.501(0.362-0.692)	<0.001
CEA (≥5ng/m vs <5ng/ml)	1.219(0.859-1.734)	0.272			
CA199 (≥27ng/m vs <27ng/ml)	1.382(0.936-1.382)	0.104			
Location					
Upper	Reference	0.281			
Middle (upper vs middle)	1.251(0.817-1.914)	0.302			
Lower (upper vs lower)	0.938(0.655-1.342)	0.725			
size (≥3cm vs <3cm)	2.230(1.567-3.174)	<0.001		0.853(0.559-1.301)	0.460
pT (yes vs no)					
T1	Reference	<0.001		Reference	0.242
T2 (T2 vs T1)	0.234((1.141-0.389)	<0.001		0.559(0.276-1.132)	0.106
T3 (T3 vs T1)	0.512(0.342-0.767)	0.001		1.105(0.687-1.778)	0.681
T4 (T4 vs T1)	0.745(0.443-1.254)	0.268		0.844(0.497-1.438)	0.534
pN					
N0	Reference	<0.001		Reference	0.004
N1 (N1 vs N0)	0.252(0.173-0.366)	<0.001		0.412(0.251-0.677)	<0.001
N2 (N2 vs N0)	0.624(0.420-0.928)	0.020		0.830(0.539-1.278)	0.397
N3 (N3 vs N0)	0.789(0.526-1.182)	0.254		0.852(0.553-1.312)	0.467
Vascular invasion (yes vs no)	2.060(1.536-2.763)	<0.001		1.030(0.700-1.517)	0.879
Perineural invasion (yes vs no)	1.569(1.179-2.087)	0.002		1.514(1.065-2.152)	0.021
Differentiated type (yes vs no)	2.039(1.460-2.848)	<0.001		0.744(0.479-1.154)	0.187
Lauren classification					
Intestinal	Reference	<0.001		Reference	0.120
Diffuse (diffuse vs intestinal)	0.407(0.279-0.594)	<0.001		0.668(0.422-1.058)	0.085
Mixed (mixed vs intestinal)	0.650(0.470-0.898)	0.009		0.730(0.515-1.034)	0.076
p53 pattern (wild-type vs mutant pattern)	1.708(1.276-2.286)	<0.001		1.652(1.220-2.238)	0.001
Locoregional recurrence (yes vs no)	2.399(1.627-3.538)	<0.001		0.37(0.246-0.576)	<0.001
Peritoneal recurrence (yes vs no)	4.000(2.819-5.921)	<0.001		0.416(0.280-0.620)	<0.001
Distant metastasis (yes vs no)	5.095(3.951-6.570)	<0.001		0.242(0.171-0.343)	<0.001
Postoperative chemotherapy (yes vs no)	4.033(2.979-5.459)	0.001		1.325(0.885-1.983)	0.172

**Abbreviation CEA:** carcinoembryonic antigen**; CA199:** Carbohydrate antigen 199.

**Table 8:** Cox regression analysis for distant metastasis of advanced-stage gastric cancer(n=301).

Clinical characteristics	Univariate analysis			Multivariate analysis	
	HR (95% CI)	P-value		HR (95% CI)	P-value
Gender (male vs female)	0.894(0.530-1.508)	0.675			
Age (≥60 yrs vs <60 yrs)	1.136(0.386-0.970)	0.037			
CEA (≥5ng/m vs <5ng/ml)	1.912(0.731-2.079)	0.433			
CA199 (≥27ng/m vs <27ng/ml)	1.163(0.650-2.083)	0.611			
Location					
Upper	Reference	0.815			
Middle (upper vs middle)	1.107(0.553-2.216)	0.775			
Lower (upper vs lower)	1.208(0.669-2.182)	0.531			
Tumor size (≥3cm vs <3cm)	0.876(0.481-1.596)	0.665			
pT (yes vs no)					
T2	Reference	0.761			
T3 (T3 vs T2)	1.011((0.552-1.851)	0.972			
T4 (T4 vs T2)	0.712(0.285-1.7781)	0.466			
pN					
N0	Reference	0.294			
N1 (N1 vs N0)	0.755((0.394-1.449)	0.398			
N2 (N2 vs N0)	0.605(0.296-1.237)	0.169			
N3 (N3 vs N0)	1.181(0.671-2.080)	0.564			
Vascular invasion (yes vs no)	1.793(1.030-4.136)	0.039		1.78 (1.022-3.099)	0.042
Perineural invasion (yes vs no)	1.322(0.829-2.107)	0.241			
Differentiated type (yes vs no)	0.548(20.238-1.264)	0.158			
Lauren classification					
Intestinal	Reference	0.206			
Diffuse (diffuse vs intestinal)	0.487(0.217-1.092)	0.081			
Mixed (mixed vs intestinal)	0.965(0.594-1.569)	0.886			
p53 pattern (wild-type vs mutant pattern)	0.624(0.394-0.988)	0.044		0.629 (0.397-0.996)	0.048
Adjuvant chemotherapy	2.898(1.169-7.186)	0.052			

**Abbreviation CEA:** carcinoembryonic antigen**; CA199:** Carbohydrate antigen 199.
